# Fractal-Based Hybrid Cryptosystem: Enhancing Image Encryption with RSA, Homomorphic Encryption, and Chaotic Maps

**DOI:** 10.3390/e25111478

**Published:** 2023-10-25

**Authors:** Dani Elias Mfungo, Xianping Fu

**Affiliations:** School of Information Science and Technology, Dalian Maritime University, Dalian 116026, China; danimfungo@dlmu.edu.cn

**Keywords:** image encryption, fractal, Sierpinski triangle, chaos theory, Paillier encryption

## Abstract

Protecting digital data, especially digital images, from unauthorized access and malicious activities is crucial in today’s digital era. This paper introduces a novel approach to enhance image encryption by combining the strengths of the RSA algorithm, homomorphic encryption, and chaotic maps, specifically the sine and logistic map, alongside the self-similar properties of the fractal Sierpinski triangle. The proposed fractal-based hybrid cryptosystem leverages Paillier encryption for maintaining security and privacy, while the chaotic maps introduce randomness, periodicity, and robustness. Simultaneously, the fractal Sierpinski triangle generates intricate shapes at different scales, resulting in a substantially expanded key space and heightened sensitivity through randomly selected initial points. The secret keys derived from the chaotic maps and Sierpinski triangle are employed for image encryption. The proposed scheme offers simplicity, efficiency, and robust security, effectively safeguarding against statistical, differential, and brute-force attacks. Through comprehensive experimental evaluations, we demonstrate the superior performance of the proposed scheme compared to existing methods in terms of both security and efficiency. This paper makes a significant contribution to the field of digital image encryption, paving the way for further exploration and optimization in the future.

## 1. Introduction

Security can be defined as the measures taken to protect any property from unauthorized users or threats that aim to modify, intercept, fabricate, interrupt, or destroy original data. The three fundamental goals of security that need to be maintained include confidentiality, integrity, and availability. In the digital era, security is crucial for the protection of digital data from malicious activities, threats, or unauthorized users. Digital security plays a vital role in safeguarding enterprises, private individuals, industries, and public organizations from data breaches. Protecting information from unauthorized access, use, disclosure, disruption, modification, perusal, inspection, recording, or destruction requires the implementation of special techniques and skills. Information can be conveyed or shared through various means, including text, video, image, and audio methods. The growing prevalence of using digital images for information transfer has spurred the development of novel techniques and methods to ensure robust security measures. Although various techniques, such as cryptography, including the methods used by Diffie and Hellman [1], Mandal et al. [2], Ovsutin O., and Dzhanashia K. [3] and the growing usage of chaos-based encryption methods presented by Alawida et al. [4], have been employed to protect digital images, either as standalone approaches or through their integration. Therefore, this paper specifically focuses on the integration of Paillier encryption as demonstrated by Paillier [5], RSA encryption as stated by Pareek et al. [6], chaos theory as stated by Scoli et al. in their work [7], and the fractal concept [8] as written by Goodchild M.F. and Mark D.M.

Chaos theory is the study of how complicated things behave over time. It looks at how small differences in starting conditions can lead to big changes in the way things develop. This can be seen in the studies conducted by Mfungo et al. [9,10,11]. Chaos theory has many applications in various fields, including physics, biology, economics, and cryptography, as stated by Jun M.A. in his study [12]. In the field of cryptography, chaos theory has been harnessed as a foundation for creating encryption algorithms that leverage the innate unpredictability and sensitivity to initial conditions exhibited by chaotic systems. These algorithms utilize chaotic functions or chaotic systems as fundamental elements in the encryption process, where the encryption key is derived from the chaotic behaviour of the system.

The use of chaos-based encryption techniques has shown promise in improving digital security [9]. These methods use the complex behaviour of chaotic systems to protect digital images from unauthorized access and data breaches [9]. This innovation presents opportunities for enhancing data protection in the digital era, especially during the transfer of sensitive information through digital images. As the digital landscape continues to evolve, these emerging security techniques are expected to play a crucial role in reducing risks related to data breaches and unauthorized access to digital assets, thereby strengthening overall digital security measures.

Several studies, such as those of Li et al., Al-Khasawneh et al., Zolfaghari B. and Koshiba T., Xian Y. et al., and Zhu Y. et al. [13,14,15,16,17], use chaotic maps to encrypt images after producing a good chaotic image algorithm for secure information transferred through the image. A comprehensive survey was conducted by Zolfaghari B. and Koshiba T. [15] on chaotic image encryption to identify current trends and directions for future research in this field. Although this study shows the trends and direction of future research, it does not provide the answer to challenges facing the field of chaotic image encryption. In their study [13], Li M. et al. established a relationship between plaintext and secret keys by securely selecting certain key pixels from the original image using a Henon map. The study also presents a novel approach to encryption that involves dividing the encryption process into two distinct parts. The first part entails encrypting the selected key pixel while preserving its secret position unchanged. The second part involves encrypting the remaining pixels using a combination of the hyper-chaotic Lorenz system and DNA encoding, which relies on the keys generated from the first part. Although this study seems to be suitable, it does not provide any means of encryption/decryption of coloured images, which could be important factors in practical applications.

In reference [9], a study conducted by Mfungo et al., we see that the new concept of integrating chaotic maps with the Kronecker xor product brings new insights to the field of image encryption. This study seems to be suitable for maintaining the confidentiality and integrity of information transferred by image. One limitation of this study is the requirement of large storage space for storing ciphertext as well as a device with high processing speed. To overcome the issue of processing speed, studies such as the one conducted by Al-Khasawneh M.A. et al. [14], which implements Hadoop-based MapReduce technology for file processing in image encryption, can be the answer. Wu J. and Yang B. [18] provide a robust image-encryption scheme. This scheme was proposed by combining the Henon map and sine map, which are chaotic systems, along with the concept of DNA from the field of biology. The resulting scheme demonstrated strong encryption capabilities, likely leveraging the chaotic behaviour of the Henon and sine maps, as well as incorporating the concept of DNA for additional security measures.

Chaos-based encryption methods provide certain benefits, such as a strong sensitivity to initial conditions, protection against attacks using cryptanalysis, and the possibility of fast encryption and decryption. Nevertheless, these techniques also come with certain difficulties, including the requirement for precise management of the chaotic system and vulnerability to attacks based on system identification or parameter estimation. In this paper, the authors specifically integrate RSA encryption; the chaos concept, particularly the logistic map [19] as used by Pareek N.K. et al.; and the Sierpinski triangle as constructed by Li C. et al. [20] from the fractal field, likely leveraging the unique properties of chaos theory and fractals to enhance the security of the RSA encryption process, as well as developing a novel encryption approach that synergistically combines these techniques.

In 1918, Gaston Julia conducted a study on the iteration of complex functions, as documented in reference [21]. This pioneering work laid the foundation for the exploration of non-regular geometric shapes known as fractals. Fractals, as described by Benoit Mandelbrot in 1979 and referenced in [22], are characterized by infinitely complex patterns that exhibit self-similarity across different scales. These shapes can be visualized as rough or fragmented geometries that exhibit repetitive patterns as they either grow or shrink, as defined by Mandelbrot B. [22,23]. An in-depth explanation of fractals can be found in this two-paper series by Husain A. et al. [24,25]. The inherent randomness and complexity of fractal images make them ideal candidates for image encryption in cryptosystems. The chaotic geometric nature of fractals results in the generation of sensitive and complex keys that can be used for encryption. The dynamic nature of fractals, with their ability to grow or shrink, further increases the size of the key space, making it even more challenging for adversaries to decipher encrypted messages. As a result, numerous studies have been conducted to explore the potential of fractals in cryptosystems for securing messages.

The significance of fractals in cryptosystems has been acknowledged by the US Navy, as evidenced by a published paper [26]. The paper presents a general-purpose and object-oriented approach for utilizing fractals in encryption and decryption processes. This underscores the importance of fractals as a powerful tool for enhancing the security of cryptographic systems. The image-encryption algorithm proposed in the study conducted by Ahmad M. [27] is based on integrating two types of fractal structures, namely Phoenix and lambda fractals. One-dimensional fractals are used as seed functions from a larger spectrum of fractal functions. The fusion of these fractal structures generates pseudo-random numbers with chaotic nature, which are used in the image encryption process. The study conducted by Duan C.F. et al. [28] encompasses the integration of the fractional discrete Tchebyshev moments, DNA coding, and fractal Sierpinski triangle model into image encryption. The findings from this research suggest that the proposed approach demonstrates resistance against both known-plaintext attacks and chosen-plaintext attacks. Other studies that employ the concept of fractals in image encryption include that of Abd-El-Hafiz et al. [29], which proposes a novel approach utilizing multiple fractal images for encryption; a study by Roziuvan et al. [30] that employs fractals to generate secret keys for plain-image encryption; and the study of Xian Y. et al. [16], which combines fractal sorting matrices and chaotic maps for encryption purposes. This paper, on the other hand, addresses the vulnerability of fractal-based encryption methods to chosen-plaintext attacks by incorporating the RSA algorithm as an additional layer of security. This enhancement aims to further bolster the security of the proposed approach, mitigating potential vulnerabilities and strengthening the encryption scheme against various attacks, including chosen-plaintext attacks.

A public-key cryptosystem called RSA (Rivest–Shamir–Adleman) is named after its inventors, Ron Rivest, Adi Shamir, and Leonard Adleman, as written in William H. [31]. It is based on the mathematical properties of prime numbers and provides a secure method for encrypting and decrypting data. The public key is used for encryption, while the private key is used for decryption. The encryption process involves raising the plaintext message to the power of the public key exponent and then taking the remainder when divided by a large modulus. The resulting ciphertext can only be decrypted using the corresponding private key, which involves raising the ciphertext to the power of the private key exponent and taking the remainder when divided by the same modulus. In ref. [32], the study proposes an approach that integrates RSA (Rivest–Shamir–Adleman), hyperchaotic, and zero-watermarking techniques for encrypting e-health images that are transferred through 6G mobile cellular networks. In ref. [6], Kota C. and Aissi C. discuss the potential vulnerabilities of RSA and propose an alternative approach to strengthen the keys used in RSA to address these weaknesses. Cryptanalytic techniques, such as factoring large numbers and continuous fraction algorithms of RSA, can potentially compromise the security of RSA-based encryption or key generation. The solution proposed is to use a larger number of keys when using continuous fraction algorithms. The proposed paper integrates RSA with other two techniques, which are the logistic map and the Sierpinski triangle, to bring novelty to image encryption.

In his study [5], Paillier presents a range of significant advantages that have yet to be fully explored in the domain of information security. With its remarkable property of enabling computations on encrypted data, this encryption scheme empowers organizations to process sensitive information while preserving confidentiality. Paillier homomorphic encryption offers advantages over conventional cryptographic techniques, enabling the computation of encrypted data while preserving privacy and functionality. Alaya B. et al. [33] extensively examined the current trends and challenges associated with the utilization of homomorphic encryption in the contemporary technological era. Their study encompassed a comprehensive review of various research works conducted by other scholars, highlighting the benefits and drawbacks of their proposed methodologies. Another significant contribution by Zhao C. et al. [34] demonstrated the significance of employing batch-based homomorphic encryption for verifying messages transmitted through vehicular ad hoc networks (VANETs). Meanwhile, Alanwar A. et al. [35] focused on ensuring the security of critical systems and devised an estimated protocol that could serve as a benchmark for such systems. Xia Z. et al. [36] explored the application of homomorphic encryption within cloud computing environments. Notably, recent studies in the field of image processing, like the work conducted by Zhang R. et al. [37], employed a combination of homomorphic encryption, thumbnail generation, and chaotic systems to effectively preserve the privacy of image information. Conversely, Singh C.E. and Sunitha C.A. [38] leveraged the concepts of Paillier encryption and blockchain technology to secure image data within cloud computing environments. In this paper, we utilize additive homomorphic encryption for the intermediate data ciphertext generated by the RSA algorithm. Subsequently, the results are segmented into pairs of digits and re-encrypted using secret keys from a 2D hybrid map. Finally, to further enhance security, we employ Paillier encryption once more to reduce the number of digits to less than four.

The design choices in this study are aimed at creating a hybrid cryptosystem that combines the strengths of multiple encryption techniques to enhance the security and robustness of the system. The proposed encryption methodology presents numerous advantages over the exclusive utilization of the RSA algorithm. This is due to the integration of the Paillier homomorphic encryption technique. The incorporation of this approach enhances the resilience of the scheme against potential deep-learning cryptoanalysis attacks, which could otherwise pose a significant threat when relying solely on the RSA algorithm. The use of RSA encryption and Paillier encryption also allows for secure key exchange and the encryption of both small and large data blocks. The use of chaotic maps and fractal concepts provides additional security by generating pseudo-random numbers and enhancing the complexity of the encryption keys. Thus, this paper aims to propose a robust and efficient cryptographic technique that combines the strengths of the RSA algorithm, homomorphic encryption, chaotic maps, and the fractal Sierpinski triangle for the encryption and decryption of images. The proposed approach has been successfully achieved. The encryption process effectively utilizes keys generated by a 2D hybrid chaotic map consisting of sine and logistic map functions. These keys are used in combination with additive homomorphic encryption to encrypt the elements. The cryptosystem employs randomly generated logistic cipher keys, which are utilized to generate initial parameters for the Sierpinski fractal triangle. Consequently, a unique fractal shape is generated incorporating sensitive keys, thus creating a large key space and enhancing randomness during the encryption process. For grayscale images, a single key is generated and employed for both encryption and decryption. However, for colour images, three distinct secret keys are generated sequentially, and these keys are used throughout the encryption and decryption procedures. Even a slight alteration in any of the keys would result in an incorrectly decrypted image, thereby ensuring the high security of the proposed scheme against statistical attacks, differential attacks, and brute-force attacks.

### The Contributions of This Paper Are as Follows

- The paper presents a ground-breaking approach to digital image encryption by combining the strengths of the Paillier homomorphic encryption, the RSA algorithm, chaotic maps, and the fractal Sierpinski triangle. This comprehensive and multi-method approach significantly enhances the algorithm’s resistance against attacks compared to methods that rely on one or two techniques. The integration of the well-established Paillier and RSA algorithms provides a robust foundation for data encryption, while the incorporation of chaotic maps and the fractal Sierpinski triangle introduces additional layers of complexity, rendering the decryption of image information considerably more challenging for potential attackers.
- This proposed scheme uses Paillier homomorphic encryption, the RSA algorithm, chaotic maps, and the fractal Sierpinski triangle to generate highly secure encryption keys resistant to cracking attempts. The fractal Sierpinski triangle’s self-similar properties result in the creation of irregular shapes at various scales, contributing to a vast array of key spaces and increased sensitivity to keys through the utilization of randomly selected initial points.
- The inclusion of chaotic maps and the fractal Sierpinski triangle in the encryption process enhances computational efficiency, particularly when compared with methods relying on complex mathematical functions. Chaotic maps, such as the logistic map, provide a simple yet powerful mechanism for introducing randomness, periodicity, and robustness, while the fractal Sierpinski triangle can be iteratively generated with minimal computational steps. This computational efficiency makes the proposed encryption approach highly practical for encrypting large volumes of data.
- The incorporation of the fractal Sierpinski triangle encryption layer opens up opportunities for customization and personalization. For instance, different types of fractals could be employed to generate unique encryption keys for each user, or users could have the ability to select their fractal parameters. This customization enhances the perceived security of the algorithm and adds an appealing aspect for end users.

The subsequent sections of the paper are structured as follows: Section 2 provides an in-depth overview of the preliminary techniques utilized in the study, while Section 3 elucidates the proposed methodology. Section 4 presents the experimental results and discusses their implications within the context of the proposed scheme. Section 5 is the final part of the proposed work. In this section, we summarize the most important lessons, explain why this research paper is important, and discuss what we plan to do in the future.

## 2. Preliminary Techniques

### 2.1. Chaotic Map

The logistic map [39] is a mathematical function that exhibits chaotic behaviour and is commonly used in cryptography for generating pseudo-random numbers. Mathematically, the logistic equation is expressed in Equation (1).

(1) $$x_{n+1}=bx_{n}-bx_{n}^{2}.$$

where b is the control parameter that determines the behaviour of the map, and x represents the initial condition value at iteration n. The parameter $x_{n+1}$ is the value of the variable at the next iteration, the value of b fits in 0 ˂ *b* ≤ 4, and x is any number from 0 to 1. The logistic map produces a sequence of values that can appear random and unpredictable, especially when the value of b is set within a specific range, typically between 3.57 and 4.0. The sine map [40], on the other hand, has been widely studied in the field of dynamical systems. This simple yet powerful equation, as seen in Equation (2), reveals complex patterns and changes as the parameter $A_{w}$ is adjusted. Researchers gain valuable insights into the fascinating behaviour of chaotic systems found in nature and physics. This map has been used in image encryption by several studies, as seen in the studies by Mfungo et al. [41] and Daoui A. et al. [42]. It remains a valuable tool in image encryption, whether used alone or in combination with other cryptographic methods. The logistic map’s chaotic behaviour and sensitivity to initial conditions make it an effective tool for generating pseudo-random numbers, thereby enhancing the security and randomness of the image encryption algorithm. Equation (2) below represents the sine map function.

(2) $$y_{n+1}=A_{w}\times \;\sin\left(\pi \times y_{n}\right),\;y\in [0,1],A_{w}>0.$$

### 2.2. Fractal Sierpinski Triangle

**Figure 1:**
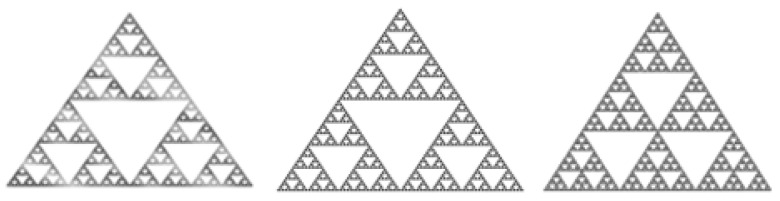
Fractal Sierpinski triangle.

According to Li C. et al. [20], the Sierpinski triangle, also known as the Sierpinski gasket or Sierpinski sieve, is a geometric shape found in nature that exhibits the essential principles of fractals. This complex shape is formed by recursively subdividing equilateral triangles into smaller ones, resulting in a repeating pattern. The Polish mathematician Waclaw Franciszek Sierpinski named this fractal along with two others, the Sierpinski carpet and Sierpinski curve, and this can be seen in the study done by Hosny K.M. et al. [43]. There are various ways to construct these triangles, such as the removal of triangles, shrinking and duplication, chaos game, arrowhead construction of the Sierpinski gasket, cellular automata, Pascal’s triangle, and towers of Hanoi [44]. Figure 1 provides an example of a fractal Sierpinski triangle.

Construction of fractal Sierpinski triangle

Let’s assume that the vertices of the initial equilateral triangle are labelled as A, B, and C, with coordinates (Ax, Ay), (Bx, By), and (Cx, Cy), respectively.

1. Base case: Draw the initial equilateral triangle.
2. Draw a line segment connecting points A, B, and C to form the initial equilateral triangle.
3. Recursive step: For each of the three sides of the equilateral triangle, divide it into three equal segments. Let’s refer to the midpoints of the segments on AB, BC, and CA as D, E, and F, respectively, with coordinates (Dx, Dy), (Ex, Ey), and (Fx, Fy), respectively.
4. Repeat the process: Apply the recursive step to each of the smaller equilateral triangles formed by the midpoints D, E, and F, using the same rule recursively.

### 2.3. Rivest–Shamir–Adleman

The RSA (Rivest–Shamir–Adleman) algorithm [6] is a widely used public key cryptographic algorithm that enhances data communication security. It operates through two main cryptographic processes. Firstly, using a public key, it transforms input data, known as plaintext, into an encrypted output referred to as ciphertext, rendering it unrecognizable without the corresponding encryption password. Retrieving the original plaintext from the ciphertext without the password in a reasonable amount of time is computationally infeasible. Secondly, utilizing a private key, the RSA algorithm can decrypt the ciphertext back into its original plaintext form, thereby completing the decryption process, as stated by Kota et al. [6]. The following are step-by-step processes of the RSA algorithm:

1. Key Generation:
  - Generate two distinct prime numbers, usually denoted as p and q.
  - Compute the modulus, n, by multiplying p and  q: n=p×q.
  - Compute Euler’s totient function, $\varphi \left(n\right),$ which is the number of positive integers less than *n* that are coprime (relatively prime) to $n:\;\varphi \left(n\right)=\left(p-1\right)\times \left(q-1\right).$
  - Choose an integer $e\;\left(1<e<\varphi \left(n\right)\right)$ as the public key exponent such that e is coprime to $\varphi \left(n\right),$ i.e., the greatest common divisor (gcd) of e and $\varphi \left(n\right),$ is 1.
  - Compute the private key exponent, d, which is the modular multiplicative inverse of e modulo $\varphi \left(n\right):$$d\equiv e^{\left(-1\right)}(\mathrm{mod}(\varphi (n)).$ This can be computed using the extended Euclidean algorithm or other modular inverse algorithms.
  - The public key is $\left(n,\;e\right),$ and the private key is $\left(n,\;d\right)$.
2. Encryption:
  - Convert the plaintext message into an integer representation, usually using a reversible encoding scheme.
  - Raise the plaintext integer to the power of e (the public key exponent) modulo n, and obtain the ciphertext: $c\equiv m^{e}(\mathrm{mod}\;n)$, where m is the plaintext integer.
3. Decryption:
  - Receive the ciphertext, c.
  - Raise the ciphertext integer to the power of d (the private key exponent) modulo n, and obtain the plaintext integer: $m\equiv c^{d}(\mathrm{mod}\;n)$.
  - Convert the plaintext integer back into the original plaintext message using the reversible encoding scheme.

### 2.4. Paillier Homomorphic Encryption

Paillier homomorphic encryption is a well-established cryptographic scheme known for its ability to perform computations on encrypted data. The core equation of Paillier encryption involves two key components: a public key $\left(n_{1},\;g\right)$ and a private key $\left(\lambda ,\;\mu \right)$. The encryption process entails raising the message m to the power of g, multiplied by a random value r, modulo $n_{1}{}^{2}$. This generates the encrypted ciphertext $c_{a}$. Homomorphic properties enable computations, such as addition and multiplication, to be carried out on ciphertexts while maintaining the integrity of the underlying plaintext. The Paillier homomorphic encryption scheme involves several parameters and operations.

1. Parameter Generation:
  - Choose two large prime numbers, $p_{1}$ and $q_{1}$.
  - Compute $n_{1}=p_{1}\times q_{1}$, which serves as the modulus for encryption and decryption.
  - Calculate $\lambda =\;\mathrm{lcm}\left(p_{1}-1,\;q_{1}-1\right),$ the least common multiple (lcm) of $\left(p_{1}-1\right)$ and $\left(q_{1}-1\right)$.
  - Select a random integer g, where $g\in Z\times n_{1}{}^{2}$ and satisfies the condition $\mathrm{mod}(g^{n_{1}},n_{1}{}^{2})=1+n_{1}$.
2. Encryption and Decryption:
  - To encrypt a message m, where $0\;\leq \;m_{1}<n_{1}$, choose a random integer r, where $r\in Z\times n_{1}$.
  - Calculate the ciphertext c using the following equation: $c=\mathrm{mod}((g^{m_{1}}\times r^{n_{1}}),n_{1}{}^{2})$.
  - Given the ciphertext c, compute the plaintext message m using the following equation: $m=((L(c_{a}{}^{\lambda }\mathrm{mod}\;n_{1}{}^{2})\times \mu )\mathrm{mod}\;n_{1})/n_{1}$.

It is important to note that the generator g, modulus $n_{1}$, and other parameters are shared publicly, while the prime numbers $p_{1}$ and $q_{1}$, as well as the private key λ, μ, are kept secret. In the decryption process, the equation involves the computation of L, which is the function defined as $L\left(x\right)=\left(x-1\right)\;/\;n_{1}$.

## 3. Design and Implementation of the Proposed Image Encryption Algorithm

In the image encryption process described, plain-image pixels are first scrambled and shuffled using predefined special keys that act as seeders, as well as randomly generated seeders and noise. The resulting scrambled image is then subjected to XOR (exclusive OR) operations using keys generated from the hybrid of a logistic map and Sierpinski triangle random keys.

### 3.1. Image Scrambling and Shuffling

**Figure 2:**
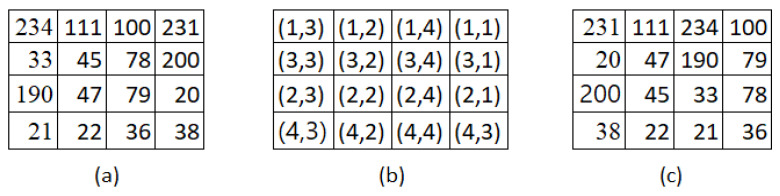
Image scrambling and shuffling. (**a**) Original pixel position, (**b**) Seed key position, (**c**) New pixel position.

A strong random seed key is generated using the system time as a seed. This key is created as a 1D array of random bytes, matching the size of the image. The image is then reshaped into a 1D array. To introduce noise or salt to the plain image, a bitwise XOR operation is applied between the reshaped image and the generated random key. This process modifies the image’s pixel values even if the values of the image are all zeros, effectively adding a layer of security or encryption to the plain image. Then, two sets, A and B, each containing random numbers from 1 to 4, are used to create a 4×4 square matrix M, as shown in Figure 2b. These numbers from the two sets are combined to generate position coordinates for relocating the pixels in the plain image P. The image P is partitioned into square matrix blocks, and each block’s pixels are scrambled based on the paired random numbers present in matrix M. Once the pixels in each block are scrambled, the blocks are combined to form matrix H. Figure 2 illustrates the process of image scrambling using 4×4 matrix blocks, while Algorithm 1 shows the scrambling pseudo-code of plain image P.
**Algorithm 1**. The pseudo-code of scrambling an image P     Input: P, Set_A, Set_B     Output: H     1. Start     2. Get image P, Set_A, Set_B.     3. $\left[M,\;N\right]=\;\mathrm{size}\left(P\right)$     4. $H=\;\mathrm{zeros}\left(M,\;N\right)$     5. rng(‘shuffle’)     6. $\mathrm{seedKey}\;=\;\mathrm{randi}\left(\left[0,\;255\right],\;1,\;\mathrm{numel}\left(P\right),\;‘uint8’\right)$     7. $P=\;\mathrm{reshape}\left(P,\;1,\;M\times N\right)$     8. $P=bitxor\left(P,\;\mathrm{seedKey}\right)$     9. for i=1:4:M
       9.1. for j=1:4:N       9.2. $\mathrm{block}\;=P\left(i:i+3,\;j:j+3\right)$         9.2.1. for x=1:4            9.2.1.1. for y =1:4            9.2.1.2. $row\_index=Set\_A\left(x\right)$            9.2.1.3. $col\_index=Set\_B\left(y\right)$            9.2.1.4. $H\left(i+x-1,\;j+y-1\right)=\;\mathrm{block}\left(row\_index,\;col\_index\right)$            9.2.1.5. end         9.2.2. end       9.3. end     10. end     11. Display H
     12. Stop

### 3.2. Encryption Using the RSA Algorithm

The randomized image H undergoes encryption using RSA algorithms to generate the encrypted image $T_{\varpi }$. In the case of coloured images, each channel is encrypted individually. The encryption process employs the RSA key, which consists of the public keys e and n. The encryption procedure follows Algorithm 2, and its implementation is depicted in Equation (3).

(3) $$T_{\varpi }=H_{}^{e}\mathrm{mod}\;(n).$$

**Algorithm 2.** The pseudo-code algorithm for the RSA encryption processInput: H, e, n
Output: $T_{\varpi }$ 1. Start 2. Get H, e, n 3. $T=\;\mathrm{zeros}\left(\mathrm{size}\left(H\right)\right)$ 4. $for\;i=1:\mathrm{size}\left(H,\;1\right)$
 4.1. $\;for\;j=1:\mathrm{size}\left(H,\;2\right)$ 4.2. $T_{\varpi }\left(i,\;j\right)=\;\mathrm{mod}\left(\mathrm{power}\left(H\left(i,\;j\right),\;e\right),\;n\right)$
 4.3. end
5. end 6. Display $T_{\varpi }$
7. Stop

### 3.3. Expansion and Reduction of Pixel Digits

The intermediary image $T_{\varpi }$ generated from RSA encryption undergoes an additional step of encryption using the Paillier encryption scheme. This process involves expanding the number of digits in each pixel position from the range of (–3) to (4–8). For example, the pixel value 205 is expanded and becomes 12,131,415. The expansion process is followed by splitting the image into four separate images, each maintaining the same size as the original. Each pair of digits is then assigned to the corresponding position in a new image. For instance, if pair 12 originates from position (123, 227) in the original image, it would be placed in image A11 at the same position (123, 227). Similarly, 13 would be assigned to image A22, 14 to image A33, and 15 to image A44, all at their respective positions.

**Figure 3:**
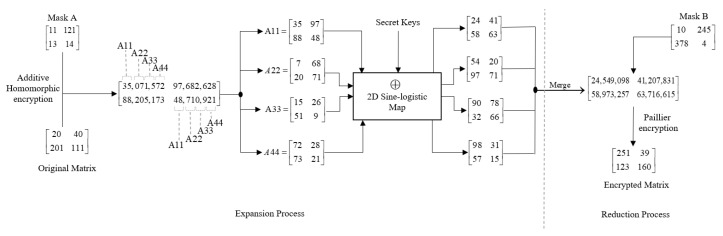
Expansion and reduction of digits.

Subsequently, each pixel in the four new images undergoes an exclusive OR (XOR) operation with the secret keys derived from a 2D sine logistic map. This XOR operation introduces additional randomness and complexity to the images. The next step involves merging the XOR-ed images to form a new image. This merged image retains the expanded digits in each pixel position, resulting from the XOR operation. To ensure privacy and facilitate further computations, the Paillier encryption scheme is applied once again to the merged image. This application of Paillier encryption reduces the expanded digit range back from 0 to 3, thus transforming the image into its final form with digits within the desired range. Figure 3 illustrates the step-by-step visualization of the expansion- and reduction-of-digits processes for a 2×2 matrix with a secret mask A.

The innovation lies in the novel combination of Paillier encryption with RSA encryption, followed by the expansion and splitting of ciphertexts, XOR operations with a 2D sine logistic map, merging of the expanded ciphertext, and final Paillier encryption, resulting in enhanced privacy, complexity, and cryptographic robustness for the encrypted image.

### 3.4. Diffusion Process by Homomorphic Paillier Additive Encryption

A partial homomorphic encryption process occurs between the image $T_{\varpi }$ and a secret Mask A, resulting in an intermediate cipher image $E_{\varpi }$. Equation (4) below is utilized to ensure that the information is securely encrypted.

(4) $$E_{\varpi }(m_{1},r_{1})\times E(m_{2},r_{2})=\left(\mathrm{mod}\left(\left(g^{m_{1}}\times r_{1}{}^{n_{1}}\right),n_{1}{}^{2}\right)\right)\times \left(\mathrm{mod}\left(\left(g^{m_{2}}\times r_{2}{}^{n_{1}}\right),n_{1}{}^{2}\right)\right),\;=\mathrm{mod}\left(\left(g^{m_{1}+m_{2}}\times {\left(r_{1}\times r_{2}\right)}^{n_{1}}\right),n_{1}{}^{2}\right).$$

such that

$$\begin{array}{l} E_{\varpi }(m_{1},r_{1})=\mathrm{mod}\left(\left(g^{m_{1}}\times r_{1}{}^{n_{1}}\right),n_{1}{}^{2}\right),\\ E_{\varpi }(m_{2},r_{2})=\mathrm{mod}\left(\left(g^{m_{2}}\times r_{2}{}^{n_{1}}\right),n_{1}{}^{2}\right). \end{array}$$

In this context, $E_{\varpi }$ represents the encrypted cipher text from $T_{\varpi }$, and secret Mask A. The $m_{1}$ and $m_{2}$ are individual pixel values of $T_{\varpi }$ and Mask A, respectively. The resulting intermediary ciphertext $E_{\varpi }$ is expanded into four sub-ciphertexts: A11,A22,A33, and A44. The sub-ciphertexts obtained from the encryption process exhibit varying numbers of digits per pixel position, typically ranging from 1 to 2. In the case of coloured images, each channel undergoes the expansion process independently, resulting in the creation of four distinct layers of sub-ciphertexts, which collectively form a grayscale image. Algorithm A1 in Appendix A has more details about the process.

### 3.5. Diffusion Process by 2D Sine Logistic Map

To enhance the security from attacks, each sub-intermediary cipher image produced by $E_{\varpi }$ undergoes exclusive operation with the secret key $Z_{x}$ obtained from Equation (5), which is the hybrid of the sine map and logistic map to form $E_{n}.$ The control parameters a, c, and f are employed to induce and manipulate the chaotic dynamics within the system. These parameters play a crucial role in generating the desired chaotic effect and shaping the behaviour of the system under study. The exclusive operation can be seen in Equation (6). All the sub-intermediaries are merged to form another intermediary ciphertext $E_{z}$, as shown in Equation (7), in which each pixel value has digits ranging from 4 to 8.

(5) $$\begin{array}{l} v_{\left(n+1\right)}=\sin(a\times z_{n})+c\times \sin(a\times v_{n})\;,\\ z_{x(n+1)}=f\times z_{n}\times (1-z_{n})+\sin(f\times v_{n})\;, \end{array}$$

(6) $$E_{n}=E_{\varpi }⊕Z_{x}\;,$$

(7) $$E_{z}{≺}_{∍}E_{n}.$$

where $A11,A22,A33,A44\in A_{n}$, and the symbol ${≺}_{∍}$ signifies the merging of all sub-ciphertexts.

### 3.6. Reducing Process by Paillier Encryption

The intermediary text $E_{z}$ undergoes an additional encryption step by applying the Paillier encryption scheme, which reduces the number of digits in each pixel value from 8 to less than 4. This reduction facilitates subsequent exclusive OR operations with the Sierpinski triangle. Notably, the private key components of the secret Mask B, namely mu $\left(\mu \right),$ lambda $\left(\lambda \right)$, and $n_{z},$ traditionally associated with private keys in other encryption schemes, are repurposed as public keys in this specific context to produce cipher text $\varpi_{\kappa }$, as shown in the mathematical Equation (8).

(8) $$\varpi_{\kappa }=\mathrm{mod}(((E_{z}{}^{\lambda })\;\mathrm{mod}(n_{z}{}^{2})-1)/n\times \mu ,n_{z}).$$

### 3.7. Diffusion Process by Logistic Map and Sierpinski Triangle

To derive distinct initial parameters for each channel, we compute the rounded modulo result of the summation of normalized pixel intensity values from the respective image channels of $\varpi_{k}$, divided by 255 and using 6 as the divisor, which is then assigned to $k_{init}$ as per Equation (9). Next, the predefined $k_{0}$ initial parameter is added to $k_{init}$ to yield a new initial parameter, $k_{i}$, as seen in Equation (10).

(9) $$k_{init}=\mathrm{round}\left(\left(\sum_{i=1}^{n}\frac{w_{k}\left(k_{i}\right)}{255}-\mathrm{floor}\left(\sum_{i=1}^{n}\frac{w_{k}\left(k_{i}\right)}{255}\right)\right)6\right),$$

(10) $$k_{i}=k_{0}+k_{init}$$

The initial values, which are the set of angles $\alpha_{\varpi }$, are evenly spaced between 8 and 10 radians and converted to Cartesian coordinates $\left(\cos\left(\alpha_{\varpi }\right),\;\mathrm{abs}\left(\sin\left(\alpha_{\varpi }\right)\right)\right)$ to determine the vertices of the Sierpinski triangle, as seen in Equation (11).

(11) $$\begin{array}{l} \alpha_{\varpi }=\;\mathrm{floor}({\left(8:10\right)}^{\prime }\times 2\times \pi /5)\;,\\ Q_{\varpi }=\left[\mathrm{abs}\left(\cos\left(\alpha_{\varpi }\right)\right),\;\mathrm{abs}\left(\sin\left(\alpha_{\varpi }\right)\right)\right]\;. \end{array}$$

We enhance the generation of coordinate patterns for the Sierpinski triangle through the utilization of secret keys generated by the logistic map. This process involves creating two sets of secret keys using two separate 1D arrays. The first array consists of secret keys s derived from the values of the logistic map with its parameter $b_{\varpi }$ and $k_{i}$, while the second array comprises secret keys $r_{\varpi }$ derived from the indices of the Sierpinski triangle’s vertices. By combining these arrays, we innovate the generation of coordinates for the Sierpinski triangle, introducing a unique and secure approach. Parameters $\mu_{\varpi }$ and $r_{\varpi }$ are generated using Equation (12).

(12) $$\begin{array}{l} \mu_{\varpi }=b_{\varpi }\times k_{i}(n)\times \left(1-k_{i}\left(n\right)\right),\\ r_{\varpi }\left(n+1\right)=\left\lceil \mu_{\varpi }\times 3\right\rceil . \end{array}$$

The generated coordinate patterns for the Sierpinski triangle, which form two columns of secret keys $c_{\omega }$, is derived by Equation (12).

(13) $$c_{\omega }(n+1,\;:)=\left(Q_{\varpi }(r_{\varpi }(n+1),\;:)+{\;c}_{\omega }(n,\;:)\right)\;/\;2.$$

To extract the long-term chaotic behaviour of the map, the initial 1000 elements are removed from $c_{\omega },$ effectively discarding the transient behaviour. Subsequently, the remaining values are converted to values within the range of 0 to 255, as seen in Equation (14), to obtain the secret keys $K_{e}.$

(14) $$K_{e}=\mathrm{floor}(255\times (1-\left\lfloor c_{\omega }(1001:\mathrm{end},1)\times (c_{\omega }(1001:\mathrm{end},2)\right\rfloor \times 0.5)).$$

To derive the ultimate cipher image $c_{f}$ as the outcome of an extensive encryption process, the intermediary cipher text $\varpi_{k}$ undergoes an exclusive OR operation with secret keys $K_{e},$ which are generated based on the modified Sierpinski triangle. By applying this method, the encryption process reaches its conclusion, ensuring the confidentiality and integrity of the cipher image. The utilization of secret keys derived from the modified Sierpinski triangle adds an extra layer of security to the encryption process, enhancing its effectiveness and resilience against unauthorized access or decryption attempts. This operation is precisely depicted in Equation (15).

(15) $$c_{f}=\varpi_{k}⊕K_{e}$$

where ⊕ denotes the XOR (exclusive OR) operation between variables. Algorithm 3 shows how secret keys are generated from the logistic–Sierpinski triangle, while Table 1 shows the summary of encryption process.
**Algorithm 3**. The pseudo-code for generating of secret keys Ke from the logistic–Sierpinski triangleInput: $b_{\varpi }$, $k_{i}$ Output: Ke 1. Start 2. $\alpha_{\varpi }=\;\mathrm{floor}((8:10)\prime \times 2\times \pi /5)$
3. $Q_{\varpi }=\left[\left(\cos\left(\alpha_{\varpi }\right)\right),\;\mathrm{abs}\left(\sin\left(\alpha_{\varpi }\right)\right)\right]$
4. $c_{\omega }\left(1,\;:\right)=\left[0,\;0\right]\;$
5. for n=1:N−1
 5.1. $\mu_{\varpi }=b_{\varpi }\times k_{i}(n)\times \left(1-k_{i}\left(n\right)\right)$ 5.2. $r_{\varpi }\left(n+1\right)=\mathrm{ceil}\left(\mu_{\varpi }\times 3\right)$ 5.3. $c_{\omega }(n+1,\;:)=\left(Q_{\varpi }(r_{\varpi }(n+1),\;:)+{\;c}_{\omega }(n,\;:)\right)\;/\;2$ 6. end
7. $K_{e}=\mathrm{floor}(255\times (1-\left\lfloor c_{\omega }(1001:\mathrm{end},1)\times (c_{\omega }(1001:\mathrm{end},2)\right\rfloor \times 0.5))$
8. Stop

### 3.8. Decryption Process

Step 1. Input the encrypted image $c_{f}.$
Step 2. Apply an exclusive operation to $c_{f}$ using secret keys $K_{e}$ derived from the logistic–Sierpinski triangle.
Step 3. Expand the number of digits by decrypting the $W_{k}$ using the decryption process for the Paillier encryption concept and utilizing the private key from Mask B.
Step 4. Split the resulting ciphertext into four sub-intermediary ciphertexts, where each ciphertext undergoes an exclusive operation with the secret keys $Z_{x}$ derived from the 2D hybrid map.
Step 5. Merge the sub-intermediary ciphertexts by concatenating the pixel values to form a reduced ciphertext $E_{w}$.
Step 6. Perform the additive homomorphic decryption process.
Step 7. Apply the RSA decryption process to the decrypted result obtained in the previous step.
Step 8. Descramble the image channels by using seed keys to restore the original images.
Step 9. Display the original image.

### 3.9. Flowchart of the Encrypted System

**Figure 4:**
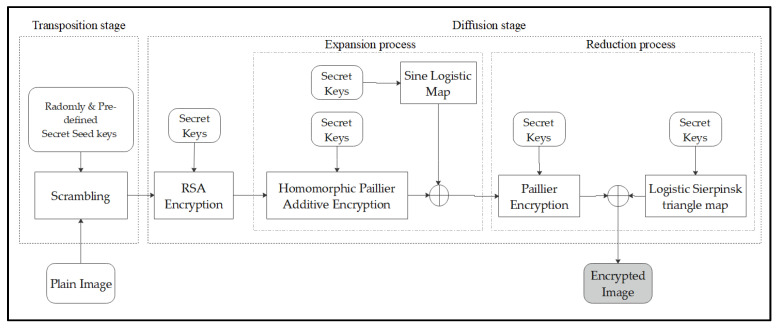
Flowchart of the proposed scheme.

Figure 4 illustrates the encryption process of the entire operation in the proposed encryption mechanism, which consists of transposition and diffusion stages.

## 4. Simulation Results and Security Analysis

### 4.1. Experimental Setup

**Figure 5:**
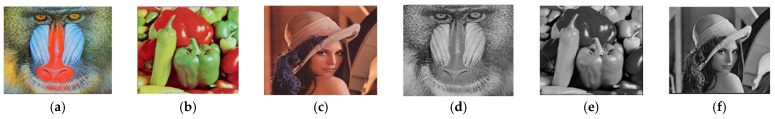
Coloured and grayscale images used for experiments. (**a**) Baboon; (**b**) Pepper; (**c**) Lena; (**d**) Baboon; (**e**) Pepper; (**f**) Lena.

In this proposed approach, we conducted tests using MATLAB R 2018a on a system with an Intel(R) Celeron^®^ CPU B820@1.70GHz, 64-bit OS, ×64 based processor, 4.0 GB RAM, and a 300 GB hard disk running Windows 10 Professional for data input. Grayscale and colourful images of varying sizes were used, as shown in Figure 5.

### 4.2. Visual Encryption and Decryption Results

**Figure 6:**
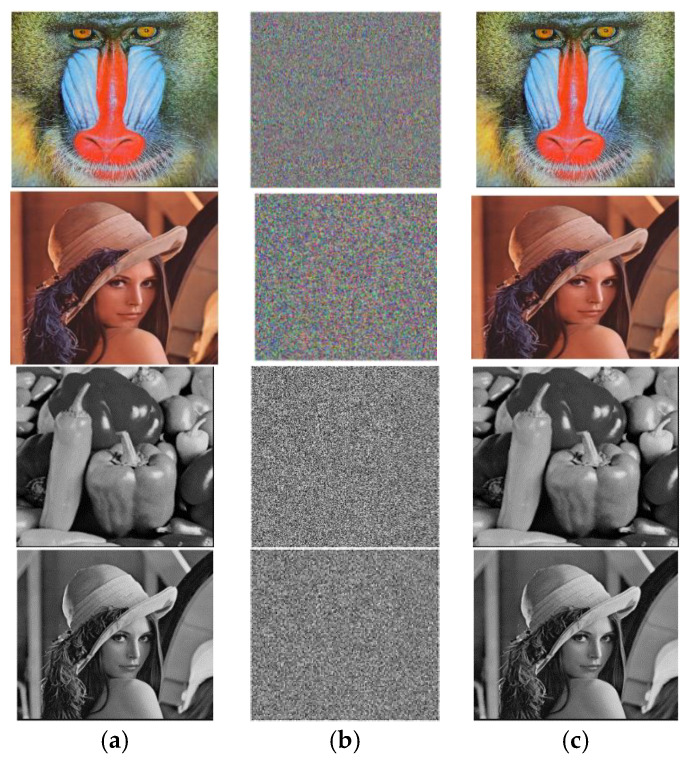
Encryption and decryption results: (**a**) Plain image; (**b**) encrypted image; (**c**) decrypted image.

Figure 6 shows the encryption and decryption findings for the images of Baboon, Pepper, and Lena in grayscale and colour of different sizes.

### 4.3. Characteristics of Nonlinear Terms in the New System’s Variations

**Figure 7:**
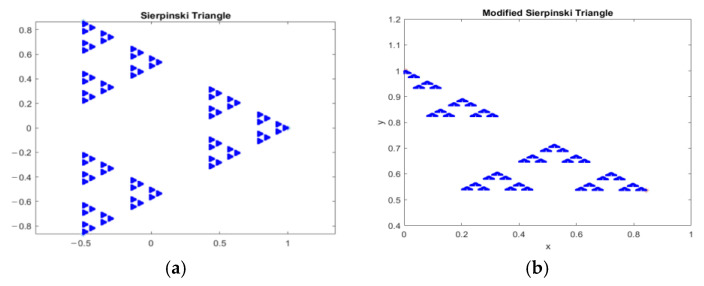
Fractal Sierpinski triangle process: (**a**) Original Sierpinski triangle; (**b**) newly generated fractal Sierpinski triangle.

**Figure 8:**
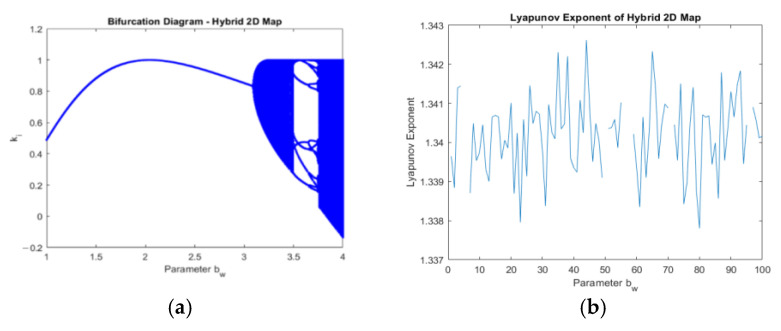
Logistic sine map: (**a**) the bifurcation diagram of the logistic sine map; (**b**) the Lyapunov exponent diagram of the logistic sine map.

The Sierpinski triangle exhibits self-similarity and a non-integer fractal dimension, while the 2D logistic sine map demonstrates a bifurcation diagram with a cascade of period-doubling bifurcations leading to chaotic behaviour, as well as complex dynamics such as periodic orbits and positive Lyapunov exponent, depending on the initial parameter values. These variation characteristics provide insight into the intricate and complex behaviour of these mathematical models and are valuable for researchers studying fractals, chaos theory, and nonlinear dynamics. Figure 7 depicts a visual diagram of the Sierpinski triangle, showcasing two scenarios: one without receiving input from a logistic map (Figure 7a), and the other with input from a logistic map (Figure 7b). The bifurcation and Lyapunov exponent diagrams are seen in Figure 8.

**Figure 9:**
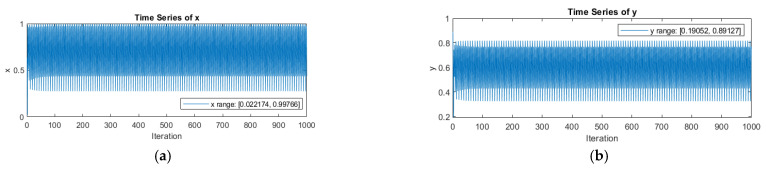
Time series diagram of 2D logistic sine map: (**a**) Time series comparison diagram with initial values (0.022174, 0.99766); (**b**) time series comparison diagram with initial values (0.19052, 0.89127).

To understand how sensitive the system is to its starting point, we analyzed two different sequences using initial values of (0.022174, 0.99766) and (0.19052, 0.89127). In Figure 9, we observe that even slight differences in the initial values can greatly impact the system’s dynamic behaviour. These findings demonstrate that the system is heavily influenced by the initial value and exhibits chaotic properties that undergo substantial changes.

### 4.4. Brute-Force Attack

#### 4.4.1. Key-Space Analysis

Key-space analysis, also referred to as key-space exploration or evaluation, is a fundamental aspect of cryptographic research involving the comprehensive analysis of the size and complexity of potential keys employed in cryptographic algorithms or systems. By carefully assessing the strength of a cryptographic key through the consideration of the number of possible keys and their distribution across the key space, key space analysis plays a crucial role in determining the security of a cryptographic scheme. Notably, a larger key space is indicative of stronger encryption, as it exponentially raises the computational effort required for an attacker to exhaustively try all possible keys in a brute-force attack. In this study, a total of eight distinct keys are utilized, each serving a unique purpose. Among these keys are the RSA secret key (e,n), the additive homomorphic encryption key $\left(n_{1},g\right)$, three keys from the 2D chaotic map (a,c,f), the Paillier public key $\left(\mu ,\lambda \right)$, and the logistic Sierpinski keys ($b_{\varpi }$, $k_{i}$). The established guidelines from IEEE [45] dictate that the standard key space should exceed $10^{15\times keys}\approx 2^{100}$, and our proposed encryption techniques meet this requirement, as confirmed by Equation (16).

(16) $$keys=\left(10^{15\times 8}\right)\;\approx {\;2}^{398}.$$

#### 4.4.2. Key Sensitive Analysis

**Figure 10:**
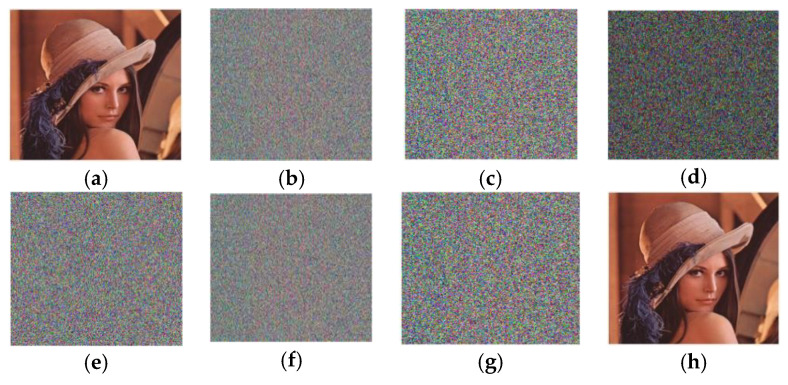
Key sensitivity analysis. (**a**) Original Image (**b**) Encrypted with $x_{0}=0.0014579\;b=3.93,$ (**c**) $\mathrm{Decipher}\;\mathrm{with}\;x_{0}=0.0014579+10^{-14}\;b=3.93,$ (**d**)$\mathrm{Decipher}\;\mathrm{with}\;x_{0}=0.0014579-10^{-14}\;b=3.93,$ (**e**) $\mathrm{Decipher}\;\mathrm{with}\;x_{0}=0.0014579\;b=3.93+10^{-14},$ (**f**) Decipher with $x_{0}=0.0014579\;b=3.93-10^{-14},$ (**g**) Decipher with $x_{0}=0.0014579-10^{-14}\;b=3.93+10^{-14},$ (**h**) Decipher with $x_{0}=0.0014579\;b=3.93$.

In a securely encrypted image, the secret keys must be extremely sensitive. Even a slight change in the secret key should produce a completely different image. The sensitivity of these secret keys is confined to the particular images employed for encryption or decryption; otherwise, unanticipated results could occur. To further enhance the sensitivity of the keys, we introduce a key code in the encryption process to ensure information secrecy. The encryption and decryption keys are updated with $\pm 10^{-14}$ to assess the key sensitivity of the proposed algorithm. This proposed scheme can perform well even if the keys are in different ranges such as $\pm 10^{-13},\pm 10^{-16},\pm 10^{-17}$. The proposed scheme employs an initial key for the logistic map, specifically $x_{0}=0.0014579$ and b=3.93. We then vary this key by making small additions or subtractions to measure the sensitivity of the encryption concerning different keys as seen in Figure 10.

### 4.5. Noise and Data-Loss Analysis

**Figure 11:**
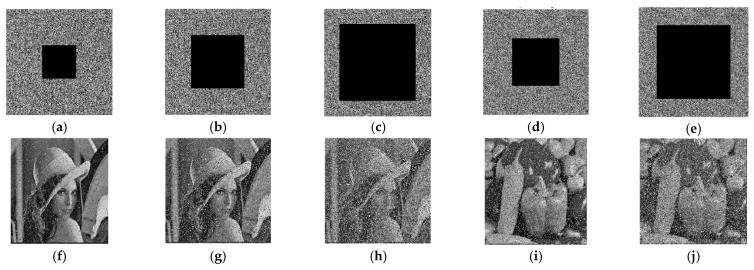
Data loss and recovery to different degrees. (**a**) Loss 10%; (**b**) Loss 35%; (**c**) Loss 50%; (**d**) Loss 25%; (**e**) Loss 45%; (**f**) Loss recovery of 10%; (**g**) Loss recovery of 35%; (**h**) Loss recovery of 50%; (**i**) Loss recovery of 25%; (**j**) Loss recovery of 45%.

**Figure 12:**
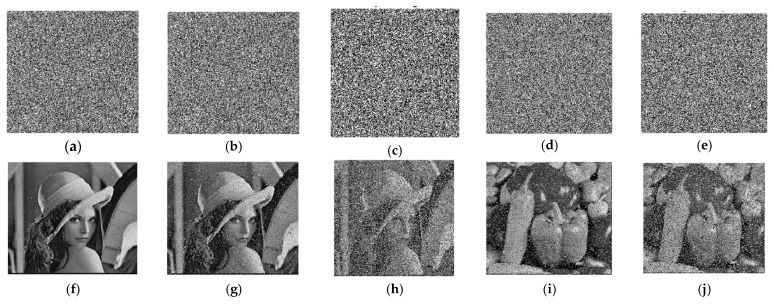
Noise effects on ciphertext and recovery images to different degrees. (**a**) Pepper and salt 0.01; (**b**) Pepper and salt 0.1; (**c**) Pepper and salt 0.5; (**d**) Pepper and salt 0.2; (**e**) Pepper and salt 0.4; (**f**) Recovery pepper and salt 0.01; (**g**) Recovery pepper and salt 0.1; (**h**) Recovery pepper and salt 0.5; (**i**) Recovery pepper and salt 0.2; (**j**) Recovery pepper and salt 0.4.

In image encryption, the presence of noise and data loss can significantly affect the quality and integrity of the information transferred by encrypted images. Therefore, it is essential to conduct a comprehensive analysis of noise and data loss in order to evaluate the performance of the encryption scheme. This analysis should include the identification of various sources of noise and data loss, the quantification of their impact on the encrypted image, and the development of appropriate techniques to mitigate their effects. To ensure the robustness and reliability of the encryption scheme, we tested noise data and data loss with different degrees. The data loss was evaluated across a range of degrees, spanning from 10% to 50% for the 256×256 image and from 25% to 45% for the 512×512 image. The proposed encryption techniques demonstrated promising results in recovering data loss up to 50% for the 256×256 image and up to 45% for the 512×512 image, as seen in Figure 11. The Lena cipher image, with a size of 256×256, and the Pepper cipher image, with a size of 512×512, were utilized as testing datasets to evaluate data loss and noise. In the 256×256 image, noise data was examined using the pepper and salt techniques. The techniques exhibit good performance in scenarios where noise was below 50% when Pepper and Salt were applied. Similarly, for the 512×512 image, the techniques demonstrated good results when pepper and salt were limited to 45%, as depicted in Figure 12.

### 4.6. Statistical Attack

One of the fundamental techniques for safeguarding information in a network is through the implementation of a digital security mechanism. This concept was pioneered by Claude Shannon, widely regarded as the “Father of Information Theory”, who introduced two key concepts for assessing the resilience of cryptographic algorithms against attacks. The assessing mechanisms are diffusion and confusion [46]. Statistical analysis, which employs methods such as histogram and correlation analysis, is commonly employed to evaluate the ability of an algorithm to withstand scrutiny and maintain robustness against such attacks.

#### 4.6.1. Correlation Coefficient Analysis

The strength of the scheme is assessed using the correlation coefficient test, which measures the degree of correlation and direction between two variables. The correlation coefficient ranges from −1 to +1, where values close to ±1 indicate a significant relationship between variables. A positive sign signifies a positive association, while a negative sign indicates a negative relationship. To meet the image security requirements, the correlation coefficient should be sufficiently low, as suggested by Zhang and Ma [47]. Mathematically, the correlation coefficient between two adjacent pixels can be represented with Equation (17), where x and y represent image pixels, E(x) represents the mean of variable x, and E(y) represents the mean variable of y.

(17) $$r_{xy}=\frac{\mathrm{cov}\left(x,y\right)}{\sqrt{\left(x\right)}\sqrt{\left(y\right)}}$$

where $\mathrm{var}(x)=\frac{1}{N}\sum_{i=1}^{N}\left(x_{i}-E{\left(x\right)}^{2}\right),\;\mathrm{cov}(x,y)=E\left(\left[x-E\left(X\right)\right]\left[y-E\left(Y\right)\right]\right),E(x)=\frac{1}{N}\sum_{i=1}^{N}x_{i},E(y)=\frac{1}{N}\sum_{i=1}^{N}y_{i}$.

**Figure 13:**
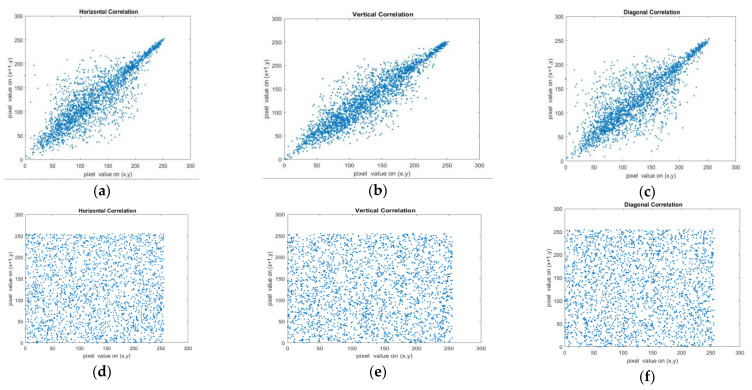
Correlations of adjacent pixels of the plain image and cipher image. (**a**) Horizontally adjacent pixels in the Baboon image’s red channel; (**b**) Vertically adjacent pixels in the Baboon image’s blue channel; (**c**) Diagonally adjacent pixels in the Baboon image’s green component; (**d**) Horizontally adjacent pixels in the red component of the encrypted image; (**e**) Vertically adjacent pixels in the blue component of the encrypted image; (**f**) Diagonally adjacent pixels in the green component of the encrypted image.

The findings reveal a range of correlation coefficients between −1 and +1. Figure 13 illustrates the pixel correlations along three directions [47]: horizontal, vertical, and diagonal of the 512×512 Baboon colour image for both the plain image and the encrypted image. In Table 2, the correlation coefficients between adjacent pixel values are presented for both grayscale and colour images of varying sizes. The plain image demonstrates coefficients that are close to 1 for all the evaluated images, indicating a strong positive relationship between adjacent pixel values. On the other hand, the encrypted image displays coefficients that are close to 0, suggesting a lack of relationship between adjacent pixel values, indicating strong encryption. The distribution of variables in each channel of the coloured images is summarized in Table 3. Additionally, Table 4 provides a comparison with other strategies in the same field, highlighting that this proposed scheme features a robust security algorithm and is more resilient to statistical attacks than other methods. The mean correlation coefficient in the positive direction for all images is 0.0008.

#### 4.6.2. Histogram Analysis

**Figure 14:**
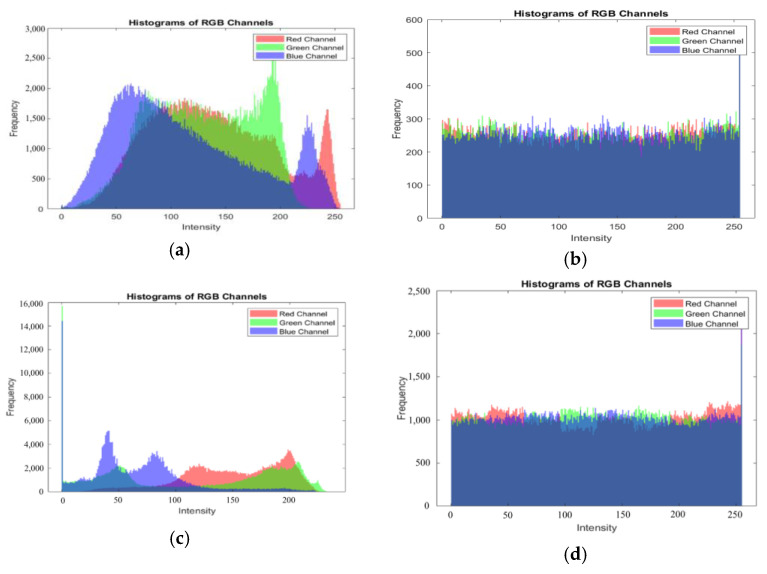
Histograms of different plain images and cipher images. (**a**) Original image of Baboon histogram (512 × 512); (**b**) Cipher image of Baboon histogram (512×512); (**c**) Original image of Pepper histogram (256 × 256); (**d**) Cipher image of Pepper histogram (256 × 256).

In image encryption, histograms are frequently employed as a means of assessing the robustness of the encryption technique. They provide a way to calculate frequency distributions for the pixel values in an image. Adversaries can launch attacks by analyzing the changes in frequency distributions in a histogram, specifically the rise and fall of these distributions. A smooth and uniform distribution is typically expected for the histogram of an encrypted image, indicating a high level of randomness and security. Compared to Zhang and Ma [47] and Zhoe et al. [51], in Figure 14, we present the histograms for both the plain and cipher images of Baboon and Pepper, showcasing the results of the proposed technique. As depicted in Figure 14, the histograms for the cipher images exhibit a uniform distribution, validating the effectiveness of the proposed technique in securely transferring information through images.

#### 4.6.3. Spatial Distribution

**Figure 15:**
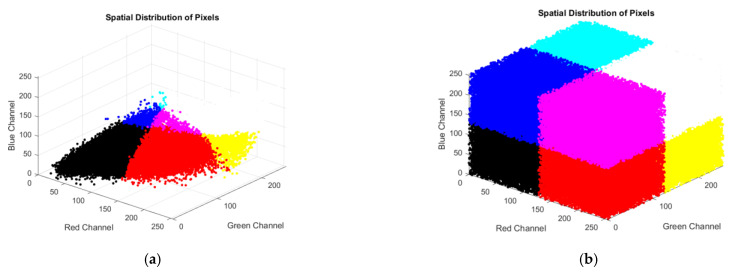
Spatial distribution of pixels in the plain image and cipher image. (**a**) Spatial distribution of pixels in the plain image; (**b**) Spatial distribution of pixels in the cipher image.

The spatial distribution of pixels in an encrypted image is a critical factor in evaluating the effectiveness of image-encryption techniques. A secure encryption process should exhibit a high degree of randomness and uniformity in the spatial distribution of pixels, which means that pixel values should be distributed evenly across the entire colour space. This randomness in the spatial distribution of pixels makes it challenging for adversaries to extract any meaningful information from the encrypted image. Additionally, a uniform spatial distribution of pixels helps to prevent the presence of any visible patterns or structures in the encrypted image, which can be exploited by attackers to launch various types of attacks, such as statistical or frequency-based attacks. In Figure 15, we show the spatial distribution of both the plain image and cipher image of Lena (512×512). Figure 15b shows a spatial distribution of pixels that is visually random, lacks any visible patterns or structures, and is uniformly distributed across the colour space, making it difficult for adversaries to deduce any meaningful information from the encrypted image.

### 4.7. Differential Attack

The utilization of metrics such as the number of pixels changing rate and the unified average changing intensity serves as an effective means to evaluate differential attacks and justify their significance. These strategies provide valuable insights into the extent of modifications introduced in images, regardless of whether they are major or minor, leading to diverse and substantial output.

#### 4.7.1. Number of Pixel Changing Rate (NPCR)

The Number of Pixel Changing Rate (NPCR) is a highly sensitive technique for detecting pixel changes in both plain and encrypted images. The NPCR is calculated as the percentage of differing pixels between two encrypted images, where a higher NPCR value indicates a greater degree of pixel variation. The ideal NPCR value is 99.609, and any scheme that approaches this value is considered well performing. If $C_{1}$ and $C_{2}$ are two encrypted images with only a one-bit difference and D represents the difference between $C_{1}$ and $C_{2}$, then the NPCR is calculated using Equation (18).

(18) $$\mathrm{NPCR}\left(C_{1}{,C}_{2}\right)=\frac{\sum_{i=1}^{M}\sum_{j=1}^{N}D\left(i,j\right)}{M\times N}\times 100\%.$$

The total size of the image is represented by M×N, and if $C_{1}\left(i,j\right)=C_{2}\left(i,j\right)$, then $D\left(i,j\right)=0,$ otherwise, $D\left(i,j\right)=1.$ Table 5 shows the NPCR values obtained for each channel in the coloured image. All NPCR values are presented in Table 6, while the comparison of our proposed system with other schemes is shown in Table 7.

#### 4.7.2. Unified Average Changing Intensity (UACI)

This is an intensity comparison between two images. It is given mathematically by Equation (19).

(19) $$\mathrm{UACI}\left(C_{1}{,C}_{2}\right)=\frac{\sum_{i=1}^{M}\sum_{j=1}^{N}\left|C_{1}\left(i,j\right)-\left.C_{2}\left(i,j\right)\right|\right.}{255\times M\times N}\times 100\%.$$

The size of the image is denoted by M×N, while $C_{1}$ and $C_{2}$ represent the encrypted images after changing one pixel by a value of $\left(\pm \right)$. A desirable value for unified average changing intensity (UACI) is close to or greater than 33.4. In this paper, all the tested values for UACI are above 33.4, by average. Table 5 displays the UACI values for each channel in the coloured image, while Table 6 presents the UACI for different images. Furthermore, the comparison of the proposed scheme with other schemes can be found in Table 7. These results highlight the effectiveness and robustness of the proposed scheme against attacks, as evidenced by the high UACI values obtained.

### 4.8. Entropy Analysis (Randomness Test)

The randomness variable in an image is measured by information entropy, mathematically given as Equation (20).

(20) $$e=\sum_{i=1}^{256}p\left(i\right)\log\left(\frac{1}{p\left(i\right)}\right).$$

When the value of e, which represents information entropy, is close to 8, it indicates that the image-encryption technique chosen is effective and meets the standards of image encryption. Table 8 and Table 9 showcase the local and global information entropy of the encrypted images, and these results are compared with those of other similar studies in Table 10. The results demonstrate that the proposed strategy for handling differential attacks is appropriate for image encryption. The high values of information entropy signify that the proposed encryption scheme effectively preserves the randomness and unpredictability of the encrypted images, making it robust against potential attacks.

### 4.9. Peak Signal-to-Noise Ratio (PSNR) and Mean Square Error (MSE) Analysis

The performance of the proposed encryption scheme is evaluated in terms of security and image quality using the peak signal-to-noise ratio (PSNR) and mean square error (MSE) analysis. A higher PSNR value signifies a lower level of distortion and better image quality, while a lower PSNR value indicates higher distortion and poorer image quality. Upon analyzing the PSNR values in Table 11 it is observed that they are relatively low, ranging from 8.1745 to 9.2931 dB, suggesting a relatively low image quality. This implies that the proposed encryption technique is effective, as it introduces a level of noise or distortion, resulting in low image quality.

Additionally, a higher MSE value indicates a larger distortion in the encrypted image. Upon analyzing the MSE values in Table 11, it is observed that they are relatively high, ranging from 100.493 to 111.147, indicating a significant level of distortion in the cipher images. This further supports the effectiveness of the proposed encryption technique, as it introduces a significant level of distortion in the encrypted images, thereby enhancing the security of the encrypted data. The PSNR and MSE values are calculated using Equations (21) and (22), respectively, in which P(i,j) represents the pixels of the original image and E(i,j) represents the pixels of the encrypted image.

(21) $$\mathrm{MSE}=\frac{1}{M\times N}\sum_{i=1}^{M}\sum_{j=1}^{N}{\left(P\left(i,j\right)-E\left(i,j\right)\right)}^{2}.$$

(22) $$\mathrm{PSNR}=10\times \log_{10}\frac{M\times N}{\sqrt{\mathrm{MSE}}}.$$

### 4.10. NIST Test

The results of 15 experimental tests, as presented in Table 12, demonstrate that the proposed encryption scheme has successfully passed the rigorous NIST (National Institute of Standards and Technology) tests for assessing the randomness of the encrypted data [52]. The *p*-values obtained for each test are notably high, ranging from 0.2757 to 0.8765, indicating that the encrypted data exhibits a high degree of randomness and does not display any statistically significant deviations from a random distribution.

### 4.11. Speed Performance Test

In this study, a speed evaluation was performed on 256×256 encrypted images, inspired by the methodology used by Yavuz [53]. To assess, a comparative analysis was conducted with previous relevant studies. Table 13 shows that the proposed encryption technique not only exhibited strong resilience but also showcased lightweight and highly efficient performance. Therefore, we firmly believe that the suggested encryption method presents a remarkable solution for achieving both secure and rapid encryption. The decryption speed is currently measured at 2.2805, which may be attributed to the laptop’s version and the software being utilized. These factors could potentially impact the overall performance. However, it is important to note that the scheme has the potential to achieve better results in a favourable environment, especially considering the rapid advancements in technology that we witness today.

### 4.12. Floating Frequency Test

**Figure 16:**
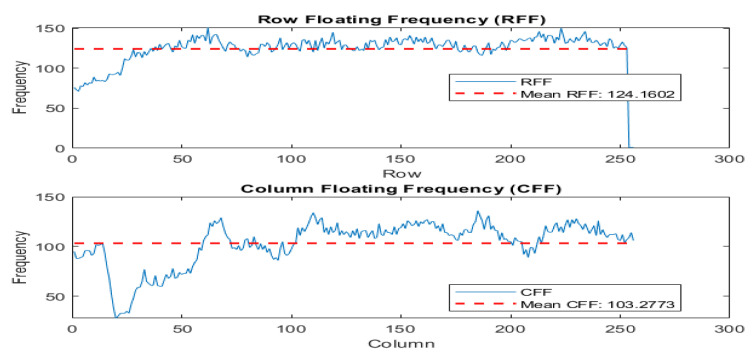
Row and column floating frequency and means for plain Lena image.

**Figure 17:**
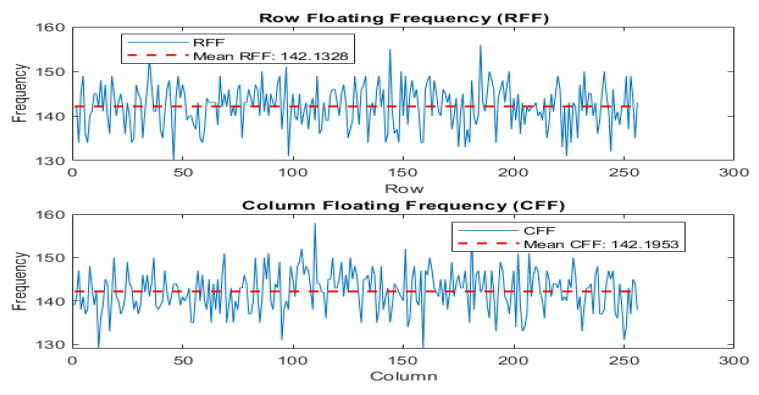
Row and column floating frequency and means for cipher Lena image.

The purpose of this test is to detect any irregularities that may indicate weaknesses in the encryption algorithm. Steps have been explained by Murillo-Escobar et al. [56]. The test involves analyzing the frequency distribution of the encrypted image to uncover potential vulnerabilities or anomalies. The test specifically examines the distribution of frequencies along the rows and columns of the image, known as the “row floating frequency” (RFF) and “column floating frequency” (CFF) tests. These tests allow for a detailed analysis of frequency characteristics in the image, considering both the horizontal and vertical directions. By evaluating the frequency distribution independently in the rows and columns, the RFF and CFF tests provide a more comprehensive evaluation of the image’s security and quality. Refer to Figure 16 and Figure 17 for visual representations of the analysis.

### 4.13. Chosen/Known Plain-Image Test

**Figure 18:**
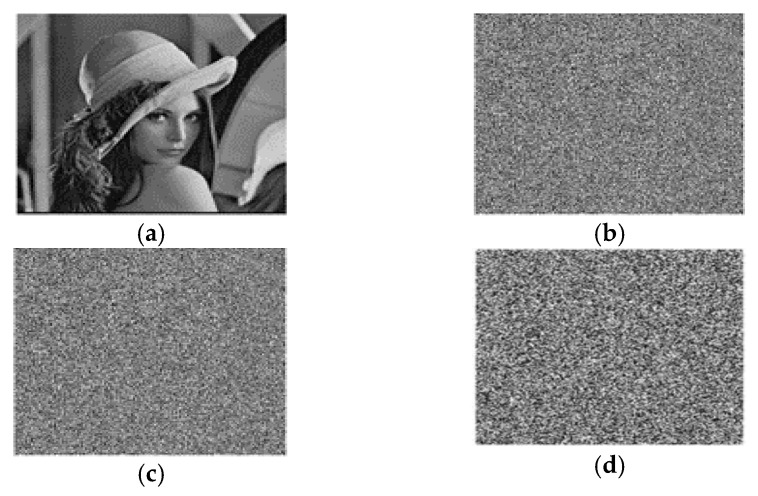
Chosen/known plain-image attack. (**a**) Chosen Lena plain image; (**b**) Encrypted Lena image; (**c**) Encrypted Pepper image; (**d**) Encrypted Pepper image with possible secret key from encrypted Lena.

The security of the image-encryption scheme is contingent upon its ability to withstand various attacks. In the case of chosen/known plain-image attacks [56], different decryption keys are employed to decrypt an encrypted image or another encrypted image. To assess the vulnerability of the algorithm to such attacks, we utilized the secret key used to decrypt the Lena image to decrypt the encrypted pepper image, as depicted in Figure 18. The decryption process fails, indicating that our algorithm is capable of effectively handling chosen/known plaintext attacks. In conclusion, the analysis demonstrates the high sensitivity of the encryption scheme to variations in decryption keys, even differences as small as $\pm 10^{-14}.$ This sensitivity makes it infeasible to utilize a secret key successfully employed for decrypting one image to decrypt another image with consistent success. For instance, a key that effectively decrypts a 512×512 Lena image may fail to decrypt a Lena image of different dimensions, such as 256×256. This underscores the robustness and security of the proposed encryption scheme against unauthorized decryption attempts using incorrect or mismatched keys.

## 5. Conclusions and Future Work

The proposed digital image-encryption technique that utilizes the pixel transposition operation, RSA algorithm, homomorphic encryption, 2D logistic sine map, and logistic fractal Sierpinski triangle is a promising approach for protecting digital images from malicious activities, threats, or unauthorized access. The scheme provides a high level of security and confidentiality for digital images, making it suitable for various applications. This encryption technique employs more than five secret keys, which enhances its effectiveness in defending against both plaintext attacks and chosen-plaintext attacks.

The experimental results show that the proposed scheme outperforms existing methods in terms of security and efficiency. The use of the RSA algorithm, Paillier encryption, 2D hybrid map, and fractal Sierpinski triangle ensure that the encryption keys are highly sensitive and difficult to crack. The self-similar properties of the fractal Sierpinski triangle produce irregular shapes at different scales, leading to a large number of key spaces and sensitivity to keys due to randomly selected initial points. The introduction of homomorphic encryption is to ensure that the system is very secure against deep-learning cryptanalysis attacks. This makes the proposed scheme suitable for handling statistical attacks, differential attacks, and brute-force attacks. Furthermore, the proposed algorithm has undergone rigorous testing using various metrics, including information entropy, PSNR (peak signal-to-noise ratio), MSE (mean squared error), and NIST (National Institute of Standards and Technology) tests, as well as assessments for noise and data loss. In all these tests, the technique has demonstrated compliance with the standards expected for secure encryption techniques.

In conclusion, the proposed scheme is characterized by its simplicity, efficiency, and high level of security, making it a valuable contribution to the field of digital image encryption. While this plan appears promising, it has a drawback: it takes a lot of time because it goes through several encryption stages. Future research could explore the potential of incorporating other chaotic maps, fractals, and traditional encryption methods such as AES, DES, and Blowfish to achieve higher and better efficiency in the field of image encryption. Other researchers can implement the algorithm on hardware platforms such as in field-programmable gate array (FPGA) and in application-specific integrated circuit (ASIC) programs to make the algorithm practical for real-world applications so as to optimize its performance and energy efficiency. Generally, this research opens up possibilities for continued exploration and advancements in the realm of digital security.

## Figures and Tables

**Table 1:** Summary of the encryption process.

Encryption Process
Input the plain image with dimensions M × N. Scramble the image using seed keys $\left(Set\_A,Set\_B\right).$ Apply the RSA algorithm to each channel of the image, resulting in the formation of ciphertext $T_{w}$. Generate two random plaintexts of size M×N, one for Mask A and the other for Mask B. Perform additive homomorphic encryption on the intermediary ciphertext $t_{w}$ using Mask A, resulting in ciphertext $E_{w}$. Split the digits of the image pixels in $E_{w}$ into pairs, forming $E_{n}.\;E_{n}$ consists of four sub-intermediary ciphertexts $\left(A11,\;A22,\;A33,\;A44\right).$ Each channel undergoes an exclusive OR operation with a secret key, $Z_{x}$, obtained from a 2D chaotic map. Concatenate all the sub-intermediary ciphertexts to form $E_{z}$. Apply the Paillier concept to reduce the resulting ciphertext to the desired range $W_{k}$. Utilize another logistic map to generate random Sierpinski triangle coordinates $K_{e}$. Use the secret keys $K_{e}$ to perform a final exclusive operation with $W_{k}$ from the Paillier encryption, resulting in the final cipher image $c_{f}$. Display the final image.

**Table 2:** Relationships among variables before and after encryption.

		Image Direction		
Image	Dimension	Horizontal	Vertical	Diagonal
Lena	256×256	0.9410	0.9143	0.9647
Encrypted Lena	256×256	−0.0041	−0.0062	0.0091
Baboon	512×512×3	0.9228	0.8547	0.8663
Encrypted Baboon	512×512×3	−0.0038	−0.0012	0.0013
Pepper	512×512×3	0.9673	0.9623	0.9684
Encrypted Pepper	512×512×3	0.0017	0.0021	0.0014
Lena	512×512×3	0.9593	0.9362	0.9717
Encrypted Lena	512×512×3	−0.0021	0.0037	0.0043

**Table 3:** Correlation coefficients of individual channels.

Image	Dimension	Channel	Image Directions		
			Horizontal	Vertical	Diagonal
Lena	256×256×3	Plain Red	0.9593	0.9362	0.9717
		Encrypted Red	0.0045	0.0017	0.0134
		Plain Green	0.9475	0.9249	0.9649
		Encrypted Green	0.0019	0.0018	0.0119
		Plain Blue	0.9222	0.8964	0.9460
		Encrypted Blue	−0.0036	−0.0027	0.0041
Baboon	256×256×3	Plain Red	0.9228	0.8547	0.8663
		Encrypted Red	0.0078	0,0045	0.0765
		Plain Green	0.8645	0.7353	0.7655
		Encrypted Green	−0.0067	−0.0004	0.0078
		Plain Blue	0.9072	0.8402	0.8810
		Encrypted Blue	0.0045	−0.0023	0.0189
Pepper	512×512×3	Plain Red	0.9673	0.9623	0.9684
		Encrypted Red	0.0057	0.0078	0.1786
		Plain Green	0.9843	0.9765	0.9863
		Encrypted Green	0.0101	0.0017	0.0245
		Plain Blue	0.9681	0.9553	0.9719
		Encrypted Blue	0.006	−0.006	0.0009

**Table 4:** Comparison of correlation coefficients.

Algorithm	Image	Direction		
		Horizontal	Vertical	Diagonal
Proposed	Lena	−0.0041	0.0062	0.0091
Hue et al. [48]		0.0004	0.0012	0.0009
Zhang et al. [47]		−0.00058	0.01091	−0.012076
Setiadi et al. [49]		−0.0011	0.0005	0.0007
Proposed	Pepper	0.0021	0.0006	0.0018
Hue et al. [48]		0.0027	0.0020	−0.0042
Alexian et al. [50]		−0.00021	0.00027	0.00128
Proposed	Baboon	−0.0003	−0.0011	0.0008
Hue et al. [48]		−0.0070	0.0021	−0.0077

**Table 5:** Values of NPCR and UACI of individual channels.

Image	Dimension	Channel	NPCR %	UACI %
Baboon	512×512	Red	99.4899	34.0123
		Green	99.6178	34.0178
		Blue	99.6235	33.0459
Pepper	512×512	Red	99.6134	34.1134
		Green	99.6052	33.9876
		Blue	99.5896	33.4359

**Table 6:** Values of NPCR and UACI for both coloured and plain cipher images.

Image	Dimension	NPCR %	UACI %
Lena	256×256	99.6601	33.6234
Lena	256×256×3	99.6521	33.8589
Pepper	256×256	99.6234	33.4709
Baboon	512×512	99.6432	33.5876
Lena	512×512×3	99.6098	33.8769
Baboon	512×512×3	99.6078	33.9062
Pepper	512×512×3	99.6187	33.4309

**Table 7:** NPCR and UACI comparison (grey images).

Image	Proposed NPCR	Hue et al. [48] NPCR	Proposed UACI	Hue et al. [48] UACI	Mfungo D.E. et al. [9] UACI
Lena	99.6601	99.62	33.6234	33.51	33.4477
Pepper	99.6234	99.64	33.4709	33.45	33.3955
Baboon	99.6432	99.59	33.5876	33.51	33.3514

**Table 8:** Information entropy of individual channels.

Image	Dimension	Channel	Local Entropy	Global Information Entropy
Baboon	512×512	Red	7.9888	7.9964
		Green	7.9994	7.9984
		Blue	7.9976	7.9961
Pepper	512×512	Red	7.9881	7.9861
		Green	7.9954	7.9968
		Blue	7.9945	7.9963

**Table 9:** Value of local and global information entropy.

Image	Dimension	Local Information Entropy	Information Entropy
Lena	256×256	7.9997	7.9979
Lena	256×256×3	7.9978	7.9985
Pepper	256×256	7.9931	7.9977
Baboon	512×512	7.9989	7.9994
Lena	512×512×3	7.9947	7.9996
Baboon	512×512×3	7.9982	7.9987
Pepper	512×512×3	7.9984	7.9989

**Table 10:** Information entropy comparison (grey images).

Image	Proposed Entropy	Zhang et al. [47] Info Entropy	Hu et al. [48] Info Entropy
Lena	7.9979	7.9994	7.9977
Pepper	7.9977	7.9994	7.9993
Baboon	7.9994	7.9994	7.9993

**Table 11:** MSE and PSNR.

	Baboon (512 × 512)	Lena (512 × 512)	Pepper (512 × 512)	Baboon (256 × 256)	Lena (256 × 256)
MSE	129.1677	112.4564	103.9954	101.569	109. 193
PSNR	8.4532	9.3425	8.2347	9.611	9.3974

**Table 12:** NIST tests.

No.	Test Name	*p*-Value	Results
1	Universal	0.4873	Success
2	Frequency	0.6783	Success
3	Block frequency	0.4003	Success
4	Cumulative sums forward	0.8963	Success
5	Cumulative sums reverse	0.7984	Success
6	Runs	0.7861	Success
7	Longest run	0.3387	Success
8	Rank	0.2018	Success
9	FFT	0.6872	Success
10	Overlapping template	0.6672	Success
11	Approximate entropy	0.6756	Success
12	Serial	0.4620	Success
13	Linear complexity	0.5782	Success
14	Random excursions	0.4981	Success
15	Random excursions variant	0.4678	Success

**Table 13:** Speed performance test.

Algorithm	Encryption Time
Proposed	1.9870
Lai and Zhang [54]	1.960
Liu et al. [55]	0.1439
Hue et al. [48]	0.480

## Data Availability

Available on request.

## Appendix A

**Algorithm A1.** The pseudo-code algorithm to expand the matrix in a sub-matrix
Input: ● Encrypted text $E_{\varpi }$ Output: ● Individual matrices **A11**, **A22**, **A33**, and **A44** 1. **Start** 2. v = zeros(size(A)); 3. for i = 1:size(A, 1) 3.1. for j = 1:size(A, 2) 3.2. num_str = num2str(A(i, j)); 3.3. num_len = length(num_str); 3.3.1. if mod(num_len, 2) ~= 0 3.3.2. % Add leading zero before the last number 3.3.3. num_str = strcat(num_str(1:end-1), ‘0′, num_str(end)); 3.3.4. end 3.3.5. if num_len < 8 3.3.6. num_str = strcat(num_str, repmat(‘0′, 1, 7—num_len)); 3.3.7. else if num_len > 8 3.3.8. % Trim the string to eight digits 3.3.9. num_str = num_str(1:8); 3.3.10. end 3.4. v(i, j) = str2num(num_str); 3.5. end 4. end 5. last_num_str = num2str(v(end)); 6. last_num_len = length(last_num_str); 7. if last_num_len < 8 8. last_num_str = strcat(last_num_str, repmat(‘0′, 1, 8—last_num_len)); 9. v(end) = str2num(last_num_str); 10. end 11. out = cellfun(@(x) reshape(x, 2, 4)’, cellstr(string(v’.*10.^(8—strlength(string(v’))))), ‘UniformOutput’, false); 12. out = cellfun(@(x) reshape(str2num(reshape(x, 2, 4)’),2,2)’, cellstr([out{:}]), ‘UniformOutput’, false); 13. A11 = out{1}; 14. A22 = out{2}; 15. A33 = out{3}; 16. A44 = out{4};
